# ﻿Phylogenomic assessment prompts recognition of the Serianthes clade and confirms the monophyly of *Serianthes* and its relationship with *Falcataria* and *Wallaceodendron* in the wider ingoid clade (Leguminosae, Caesalpinioideae)

**DOI:** 10.3897/phytokeys.205.79144

**Published:** 2022-08-22

**Authors:** Else Demeulenaere, Tom Schils, J. Gordon Burleigh, Jens J. Ringelberg, Erik J. M. Koenen, Stefanie M. Ickert-Bond

**Affiliations:** 1 Center for Island Sustainability, University of Guam, UOG Station, Mangilao, 96923, Guam; 2 Marine Laboratory, University of Guam, UOG Station, Mangilao, 96923, Guam; 3 Department of Biology, University of Florida, PO Box 118525, Gainesville, FL 32611-8525, USA; 4 Department of Systematic and Evolutionary Botany, University of Zurich, Zollikerstrasse 107, 8008 Zurich, Switzerland; 5 Evolutionary Biology & Ecology, Free University of Brussels, Av. F.D. Roosevelt, 50, CP 160/12 - B-1050 Brussels, Belgium; 6 Department of Biology & Wildlife, Herbarium (ALA) at the University of Alaska Museum of the North, University of Alaska Fairbanks, P.O. Box 757720, Fairbanks AK 99775-7720, USA

**Keywords:** Archidendron clade, Fabaceae, mimosoid clade, monophyly, phylogenomics, targeted enrichment sequencing

## Abstract

The Indo-Pacific legume genus *Serianthes* was recently placed in the Archidendron clade (sensu Koenen et al. 2020), a subclade of the mimosoid clade in subfamily Caesalpinioideae, which also includes *Acacia*, *Archidendron*, *Archidendropsis*, *Falcataria*, *Pararchidendron*, *Paraserianthes* and *Wallaceodendron*. *Serianthes* comprises ca. 18 species, five subspecies and two varieties that are characterised by bipinnately compound leaves with alternate sessile leaflets, branched axillary corymbiform panicles and woody indehiscent pods. Generic relationships, as well as species relationships within genera in the Archidendron clade, remain uncertain. While the sister relationship between *Serianthes* and the genus *Falcataria* is strongly supported by molecular data, the distinction between *Serianthes* and the monotypic genus *Wallaceodendron* has been questioned, based on their similar flower and fruit morphologies. We combined three gene-enriched hybrid capture DNA sequence datasets (generated from the 964 mimobaits v1 probe set, the expanded 997 mimobaits v2 probe set and the GoFlag angiosperm 408 probe set) and used their overlapping markers (77 loci of the target exonic and flanking regions) to test the monophyly of *Serianthes* and to investigate generic relationships within the Archidendron clade using 55 ingoid plus two outgroup taxa. We show that *Serianthes* is monophyletic, confirm the *Serianthes* + *Falcataria* sister relationship to *Wallaceodendron* and recognise this combined clade as the Serianthes clade within the Archidendron clade. We also evaluated the use of overlapping loci across datasets in combination with concordance analyses to test generic relationships and further investigate previously unresolved relationships across the wider ingoid clade. Concordance analysis revealed limited gene tree conflicts near the tips of the Archidendron clade, but increased discordance at the base of the clade, which could be attributed to rapid lineage divergence (radiation) and/or incomplete lineage sorting.

## ﻿Introduction

**Figure 1.**
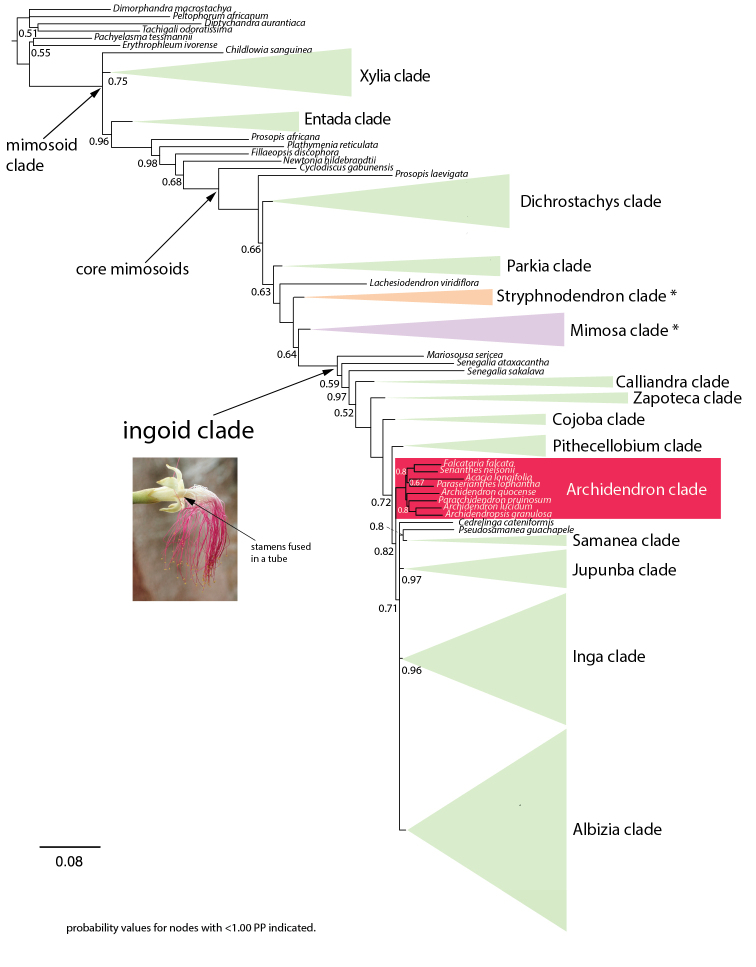
Phylogeny of the mimosoid clade modified from Koenen et al. (2020), based on the mimosoid 964 nuclear dataset with the Archidendron clade highlighted in red. Clade names follow Koenen et al. (2020) with branches collapsed and represented by green triangles. The Stryphnodendron and Mimosa clades, taxa from which were used to root trees in this study, are highlighted in orange and purple, respectively and indicated with an asterisk.

In the recent re-classification of legume subfamilies (LPWG 2017), the former subfamily Mimosoideae that is nested within the re-circumscribed Caesalpinioideae, was informally recognised as the mimosoid clade. Within the mimosoid clade, phylogenetic analyses (e.g. Luckow et al. 2003; Bruneau et al. 2013; LPWG 2017; Koenen et al. 2020) consistently show that none of the tribes in the traditional tribal classification of Bentham (1844) are monophyletic. Recent phylogenomic analyses provided greater resolution across the mimosoid phylogeny (Koenen et al. 2020) and Caesalpinioideae as a whole (Ringelberg et al. 2022), establishing the basis for the recognition of a number of informally-named clades, including the large pantropical ingoid clade (Koenen et al. 2020) that contains all genera of tribe Ingeae plus *Acacia* Mill. and all its segregates, except *Vachellia* Wight & Arn. Morphologically, this clade is characterised by flowers with > (10–)30 stamens that are often fused into a tube (Fig. 1; Brown et al. 2008; Koenen et al. 2020).

**Figure 2.**
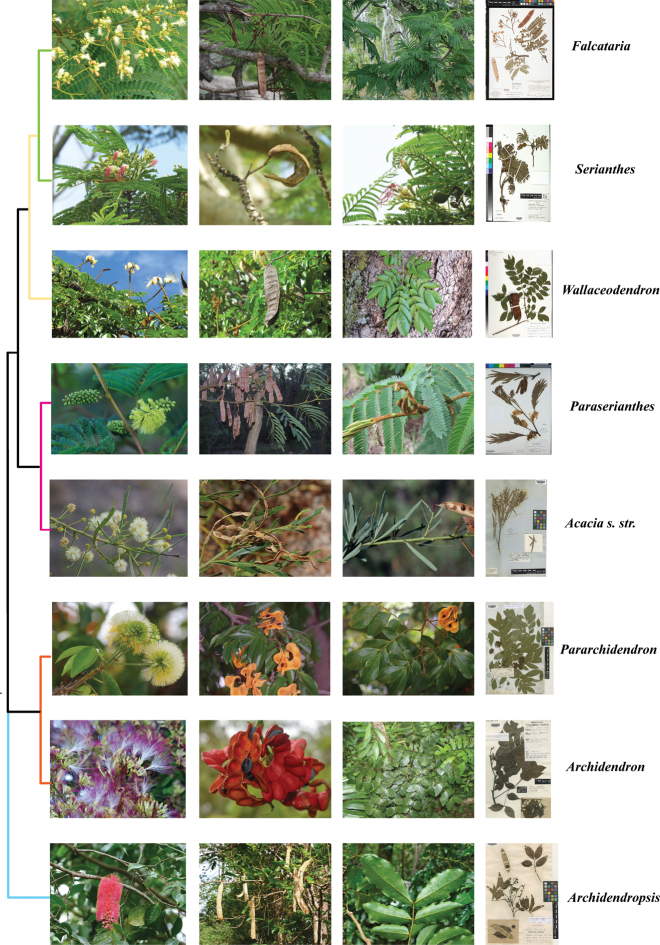
Morphology and relationships of the genera of the Archidendron clade, based on relationships recovered in our ASTRAL analysis. The colour scheme follows that in Fig. 7. Images are used with permission from Flickr: *Acaciarostellifera* (PC: Russell Cumming, HS: K000779891), *Archidendrongrandiflorum* (PC: fl, le: Russell Cumming, HS: K000724398), *Archidendronlucyi* (PC: fr: Russell Cumming), Archidendropsispaivanasubsp.balansae I.C. Nielsen (PC: fl: Benoît Henry), *Archidendropsisstreptocarpa* (Fournier) I.C. Nielsen (PC: fr, le: Benoît Henry, HS: K000822329), *Falcatariafalcata* (Photo credits [PC]: flower [fl]: JB Friday), *Falcatariatoona* (PC: fruit [fr], leaf [le]: Russell Cumming, herbarium sheet [HS]: NY0149795, *Serianthesnelsonii* (PC: Else Demeulenaere, HS: US00689615), *Pararchidendronpruinosum* (PC: Russell Cumming, HS: K000759556), *Paraseriantheslophantha* (PC: fl: Eric Hunt; fr: Russell Cumming, le: Forest Starr and Kim Starr, HS: OBI126697), *Wallaceodendroncelebicum* (PC: Plantaholic Sheila, HS: LSU00096994).

Koenen et al. (2020) found that the Indomalayan/Australasian Archidendron clade falls within the ingoid clade (Fig. 1). The Indo-Pacific genus *Serianthes* Benth., which is the focus of this study, is included in the Archidendron clade, together with seven other genera (Koenen et al. 2020; Table 1, Fig. 2): *Acacia* s.s., *Archidendron* F. Muell., *Archidendropsis* I.C. Nielsen, *Falcataria* (I.C. Nielsen) Barneby & J.W. Grimes, *Pararchidendron* I.C. Nielsen, *Paraserianthes* I.C. Nielsen and *Wallaceodendron* Koord. The Archidendron clade is restricted to the Indomalayan and Australasian realms, with highest species diversity and endemism in Malesia, Papua New Guinea, New Caledonia and Australia (Table 1).

**Table 1.** Genera of the Archidendron clade: diversity, distribution and sampling included in the current study.

Genus	# of spp.	Distribution	# of spp. incl.	Literature Cited
***Acacia* Mill. s.s.**	986–1045	Mostly from Australia incl. 19 phyllodinous spp. from Hawai‘i to Madagascar	3	Brown et al. (2008); Koenen et al. (2020)
***Archidendron* F. Muell.**	96	Endemic to SE Asia, the Pacific Islands and Australia	3	Fosberg (1960); Brown et al. (2008, 2010); Koenen et al. (2020)
***Archidendropsis* I.C. Nielsen**	11	Endemic to northern Australia (Queensland), New Caledonia, the Bismarck Archipelago and New Guinea	2	Nielsen et al. (1983, 1984a, 1984b); Brown et al. (2011); Koenen et al. (2020)
***Falcataria* (I.C. Nielsen) Barneby & J.W. Grimes**	3	Endemic to SE Asia, Papua New Guinea, the Solomon Islands and Australia	1	Brown et al. (2011); Koenen et al. (2020)
***Pararchidendron* I.C. Nielsen**	1, two subspecies and one variety	Java, Saleier Island, Bali, Lombok, Sumba, Sumbawa, Flores, Timor, Papua New Guinea and Australia (Queensland & New South Wales)	1	Nielsen et al. (1983, 1984a, 1984b); Brown et al. (2011); Koenen et al. (2020)
***Paraserianthes* I.C. Nielsen**	1	Java, Sumatra, the Lesser Sunda Islands and Australia	1	Nielsen et al. (1983, 1984a, 1984b); Brown et al. (2011); Koenen et al. (2020)
***Serianthes* Benth.**	18	Indo-Pacific Region	8	Nielsen et al. (1983, 1984a, 1984b); Koenen et al. (2020)
***Wallaceodendron* Koord.**	1	North Sulawesi and the Philippines	1	Nielsen et al. (1983, 1984, 1984a, 1984b); Brown et al. (2011)

Nielsen (1992) and Nielsen et al. (1983, 1984a, 1984b) solidified the classification of the genera in the Archidendron clade and this generic system is still largely followed today. However, apart from *Acacia* s.s. (Brown et al. 2008), the monophyly of most of the ingoid genera in the Archidendron clade has not been tested with modern phylogenetic and phylogenomic analyses until recently (Brown et al. 2022; Ringelberg et al. 2022). Recent efforts to resolve phylogenetic relationships within the species-rich Archidendron clade have been hampered by a paucity of molecular data or incomplete taxon sampling in previous studies (Brown et al. 2008; Brown et al. 2011; Koenen et al. 2020). These uncertainties are compounded by nomenclatural instability (Barneby and Grimes 1996; Brown et al. 2008), lack of fertile herbarium specimens and morphological homoplasy (Fosberg 1960; Nielsen et al. 1984a; Koenen et al. 2020), as well as extensive geographic ranges for some species spanning the Indo-Pacific and Australia (Strijk et al. 2020). In the age of museomics and collection-based phylogenomics, the ability to sequence DNA from historical museum specimens (Zedane et al. 2016; Moreno-Aguilar et al. 2020; Renner et al. 2021) provides new opportunities to analyse phylogenetic relationships within the species-rich Archidendron clade by expanding taxon sampling geographically and including expert-identified specimens. Targeted enrichment sequencing (e.g. Hyb-Seq) can generate phylogenomic data by extracting DNA from small amounts of leaf tissue from archived herbarium specimens to build phylogenies with greatly enhanced gene and taxon representation (Bossert and Danforth 2018; Johnson et al. 2019; Escudero et al. 2020; Bateman et al. 2021; Eriksson et al. 2021).

*Serianthes* is a genus of tropical trees and shrubs distributed in the Indo-Pacific (Southeast Asia, the Pacific Islands and Australia). The genus was described by Bentham (1844) and has been revised by Fosberg (1960) and Kanis (1979, only the Malesian species). The most recent revision of *Serianthes* (Nielsen et al. 1984b) recognised 18 species, five subspecies and four varieties. The infrageneric classification of Nielsen et al. (1984a) recognised two subgenera, based on the basic unit of the inflorescence, subgenus Minahassae Fosberg with racemosely arranged pedunculate spikes and subgenus Serianthes with racemosely arranged pedunculate racemes, umbels or glomerules, while pod dehiscence and pod valve morphology were used to define sections within subgenus Serianthes. Although the monophyly of *Serianthes* has not been questioned, certain *Albizia* and *Acacia* taxa have been transferred to *Serianthes* in taxonomic revisions (Fosberg 1960).

Most *Serianthes* species are island endemics confined to small archipelagos in the Indo-Pacific Ocean. These endemic species face varying degrees of extinction threat caused by habitat loss and spread of invasive species. The IUCN Red List of Threatened Species lists 12 species of *Serianthes*, with three designated as critically endangered (IUCN 2021). In addition, *Serianthesnelsonii* Merr., endemic to the Mariana Islands, Guam and Rota, is listed as critically endangered by the U.S. Endangered Species Act (U.S. Fish and Wildlife Service 1987); only a single mature tree remains in Guam (Indigenous name [IN] for *S.nelsonii* on Guam: Håyun Lågu) and fewer than 50 individuals on Rota (IN: Tronkon Guåfi). As traditional uses and endemic languages are intrinsically connected to these endemic species, the islands’ biocultural diversity is also vulnerable to extinction. Indigenous island communities traditionally use *Serianthes* trees for building canoes, boats and meeting houses, as ethnomedicines, in agriculture and in handicrafts (Demeulenaere et al. 2021).

Nielsen et al. (1983, 1984a, 1984b) discussed the generic limits of *Serianthes* and the other Malesian, Australian and Pacific Ingeae, based on comparative morphology. Nielsen et al. (1983) considered *Serianthes* to be closely related to *Falcataria* (as *Paraserianthesfalcataria*) and *Wallaceodendron*, based on their wood anatomy and postulated that they were more closely related to the group of *Paraserianthes* s.s., *Archidendropsis* and *Pararchidendron* than to *Archidendron*. The eophylls of *Falcataria* and *Serianthes* are bipinnate, while all other genera in the Archidendron clade have once-pinnately compound eophylls (Nielsen et al. 1983). In 1996, Barneby and Grimes established *Falcataria* as a new genus, based on Nielsen’s ParaserianthessectionFalcataria, which included three species. This treatment was validated by the phylogenetic study of Brown et al. (2011), which concluded that *Paraserianthes* was paraphyletic and provided strong evidence for a well-supported *Falcataria* clade (incl. *Falcatariafalcata* (L.) Greuter & R. Rankin, *Falcatariapullenii* (Verdc.) Gill. K. Br. and *Falcatariatoona* (F.M. Bailey) Gill. K. Br., D.J. Murphy & Ladiges), distinct from *Paraseriantheslophantha* (Willd.) I.C. Nielsen of Nielsen’s ParaserianthessectionParaserianthes.

A recent phylogenomic study of the mimosoid clade included seven of the eight genera of the Archidendron clade (Koenen et al. 2020) and was the first study to include one of the 18 species of *Serianthes*, but it did not sample the monotypic *Wallaceodendron*. Here, we used data from targeted sequence capture to evaluate the monophyly of *Serianthes* by combining a large dataset for mimosoid legumes (Koenen et al. 2020) with a separate phylogenomic dataset for *Serianthes* and genera of the Archidendron clade.

## ﻿Methods

### ﻿Sampling

We used sequences generated from three target capture probe sets: 1) The Mimobaits probe set v1 including 964 nuclear genes of Koenen et al. (2020; https://github.com/erikkoenen/mimobaits/), 2) the Mimobaits probe set v2 (expanded from v1 including 997 nuclear genes, Ringelberg et al. 2022) and 3) the GoFlag angiosperm 408 probe set which includes 408 nuclear exons and their flanking regions (Breinholt et al. 2021a). Merging these datasets resulted in alignments with 57 taxa of the ingoid clade and outgroups, of which 19 belong to the Archidendron clade (Tables 1, 2). Eight of the 18 species of *Serianthes* were included, covering the distribution range of the genus and members of both subgenera and the two sections in subgenus Serianthes. Outgroup selection followed previous phylogenies of mimosoid legumes (Koenen et al. 2020) to select *Stryphnodendronpulcherrimum* Hochr. and *Mimosagrandidieri* Baill. as the outgroup.

**Table 2.** Sample information for the taxa included in the ingoid clade phylogeny. This table includes sampling code/accession and voucher information for 57 taxa with the herbarium acronym shown in parentheses, dataset name and publication. Taxa belonging to the Archidendron clade are indicated with an asterisk.

Species	Accession	Voucher	Database	Publication
***Abaremacochliacarpos* (Gomes) Barneby & J.W. Grimes**	ERS4812838	L.P. de Queiroz 15538 (HUEFS)	mimosoid 964 nuclear dataset	Koenen et al. (2020)
*Acaciarostellifera* Benth.*	ERS11697109	Murphy 466 (MELU)	expanded mimosoid 977 nuclear dataset	Ringelberg et al. (2022)
***Acaciavictoriae* Benth.** *	ERS11697114	Ariati 260 (MELU)	expanded mimosoid 977 nuclear dataset	Ringelberg et al. (2022)
***Albiziaadianthifolia* (Schumach.) W. Wight**	ERS4812846	J.J. Wieringa 6278 (WAG)	mimosoid 964 nuclear dataset	Koenen et al. (2020)
***Albiziaaltissima* Hook.f.**	ERS4812847	C. Jongkind 10709 (WAG)	mimosoid 964 nuclear dataset	Koenen et al. (2020)
***Albiziaatakataka* Capuron**	ERS4812849	E. Koenen 229 (Z)	mimosoid 964 nuclear dataset	Koenen et al. (2020)
***Albiziaaurisparsa* (Drake) R. Vig.**	ERS4812850	E. Koenen 230 (Z)	mimosoid 964 nuclear dataset	Koenen et al. (2020)
***Albiziaferruginea* (Guill. & Perr.) Benth.**	ERS4812857	C. Jongkind 10762 (WAG)	mimosoid 964 nuclear dataset	Koenen et al. (2020)
***Albiziagrandibracteata* Taub.**	ERS4812858	E. Koenen 159 (WAG)	mimosoid 964 nuclear dataset	Koenen et al. (2020)
***Albiziainundata* (Mart.) Barneby & J.W. Grimes**	ERS4812859	J.R.I. Wood 26530 (K)	mimosoid 964 nuclear dataset	Koenen et al. (2020)
***Albiziamahalao* Capuron**	ERS4812860	E. Koenen 216 (Z)	mimosoid 964 nuclear dataset	Koenen et al. (2020)
***Albiziamasikororum* R. Vig.**	ERS4812861	E. Koenen 237 (Z)	mimosoid 964 nuclear dataset	Koenen et al. (2020)
***Albiziaobbiadensis* (Chiov.) Brenan**	ERS4812862	Thulin 4163 (UPS)	mimosoid 964 nuclear dataset	Koenen et al. (2020)
***Albiziaobliquifoliolata* De Wild.**	ERS4812863	J.J. Wieringa 6519 (WAG)	mimosoid 964 nuclear dataset	Koenen et al. (2020)
***Albiziaretusa* Benth.**	ERS4812865	Hyland 2732 (L)	mimosoid 964 nuclear dataset	Koenen et al. (2020)
***Albiziasahafariensis* Capuron**	ERS4812866	E. Koenen 405 (Z)	mimosoid 964 nuclear dataset	Koenen et al. (2020)
***Albiziasaponaria* (Lour.) Blume**	ERS4812867	Jobson 1041 (BH)	mimosoid 964 nuclear dataset	Koenen et al. (2020)
***Albiziaumbellata* (Vahl) E.J.M. Koenen**	ERS4812882	Jobson 1037 (BH)	mimosoid 964 nuclear dataset	Koenen et al. (2020)
***Albiziaversicolor* Welw. ex Oliv**,	ERS4812868	O. Maurin 560 (JRAU)	mimosoid 964 nuclear dataset	Koenen et al. (2020)
***Albiziaviridis* E. Fourn.**	ERS4812869	Du Puy M251 (K)	mimosoid 964 nuclear dataset	Koenen et al. (2020)
***Albiziazygia* (DC.) J.F. Macbr.**	ERS4812870	J.J. Wieringa 5915 (WAG)	mimosoid 964 nuclear dataset	Koenen et al. (2020)
***Archidendrongrandiflorum* (Soland. ex Benth.) I.C. Nielsen** *	ERS11697138	Clarkson 6233 (L)	expanded mimosoid 977 nuclear dataset	Ringelberg et al. (2022)
***Archidendronlucidum* (Benth.) I.C. Nielsen** *	ERS4812873	Wang and Lin 2534 (L)	mimosoid 964 nuclear dataset	Koenen et al. (2020)
***Archidendronquocense* (Pierre) I.C. Nielsen** *	ERS4812874	Newman 2094 (E)	mimosoid 964 nuclear dataset	Koenen et al. (2020)
***Archidendropsisgranulosa* (Labill.) I.C. Nielsen** *	ERS4812875	McKee 38353 (L)	mimosoid 964 nuclear dataset	Koenen et al. (2020)
***Archidendropsisxanthoxylon*** *	ERS11697143	Hyland 9229 (L)	expanded mimosoid 977 nuclear dataset	Ringelberg et al. (2022)
***Baliziapedicellaris* (DC.) Barneby & J.W. Grimes**	ERS4812877	L.P. de Queiroz 15529 (HUEFS)	mimosoid 964 nuclear dataset	Koenen et al. (2020)
***Balizia* sp.nov.**	ERS4812878	M.P. Morim 577 (RB)	mimosoid 964 nuclear dataset	Koenen et al. (2020)
***Blanchetiodendronblanchetii* (Benth.) Barneby & J.W. Grimes**	ERS4812879	L.P. de Queiroz 15616 (HUEFS)	mimosoid 964 nuclear dataset	Koenen et al. (2020)
***Chloroleucontenuiflorum* (Benth.) Barneby & J.W. Grimes**	ERS4812885	L.P. de Queiroz 15514 (HUEFS)	mimosoid 964 nuclear dataset	Koenen et al. (2020)
***Cojobaarborea* (L.) Britton & Rose**	ERS4812886	M.F. Simon 1545 (CEN)	mimosoid 964 nuclear dataset	Koenen et al. (2020)
***Falcatariafalcata* (L.) Greuter & R. Rankin**	ERS4812898	Ambri & Arifin W826A (K)	mimosoid 964 nuclear dataset	Koenen et al. (2020)
***Havardiapallens* (Benth.) Britton & Rose**	ERS4812900	C.E. Hughes 2138 (FHO)	mimosoid 964 nuclear dataset	Koenen et al. (2020)
***Hesperalbiziaoccidentalis* (Brandegee) Barneby & J.W. Grimes**	ERS4812901	C.E. Hughes 1296 (FHO)	mimosoid 964 nuclear dataset	Koenen et al. (2020)
***Hydrochoreacorymbosa* (Rich.) Barneby & J.W. Grimes [2**]	ERS4812903	J.R. Iganci 862 (RB)	mimosoid 964 nuclear dataset	Koenen et al. (2020)
***Jupunbatrapezifolia* (Willd.) Britton & Killip**	ERS4812839	M.F. Simon 1600 (CEN)	mimosoid 964 nuclear dataset	Koenen et al. (2020)
***Leucochloronbolivianum* C.E. Hughes & Atahuachi**	ERS4812907	C.E. Hughes 2608 (FHO)	mimosoid 964 nuclear dataset	Koenen et al. (2020)
***Leucochloronlimae* Barneby & J.W. Grimes**	ERS4812908	MWC8250 (K)	mimosoid 964 nuclear dataset	Koenen et al. (2020)
***Mariosousasericea* (M. Martens & Galeotti) Seigler & Ebinger**	ERS4812911	MWC18949 (K)	mimosoid 964 nuclear dataset	Koenen et al. (2020)
***Mimosagrandidieri* Baill.**	ERS4812912	E. Koenen 207 (Z)	mimosoid 964 nuclear dataset	Koenen et al. (2020)
***Pararchidendronpruinosum* (Benth.) I.C. Nielsen** *	ERS4812919	Jobson 1039 (BH)	mimosoid 964 nuclear dataset	Koenen et al. (2020)
***Paraseriantheslophantha* (Willd.) I.C. Nielsen** *	ERS4812920	M. van Slageren & R. Newton MSRN648 (K)	mimosoid 964 nuclear dataset	Koenen et al. (2020)
***Pithecellobiumdulce* (Roxb.) Benth.**	ERS4812927	B. Marazzi 309 (ASU)	mimosoid 964 nuclear dataset	Koenen et al. (2020)
***Samaneasaman* (Jacq.) Merr.**	SRR18455122	Demeulenaere E, GUAM	GoFlag 408 dataset	This contribution
***Senegaliaataxacantha* (DC.) Kyal. & Boatwr.**	ERS4812938	C. Jongkind 10603 (WAG)	mimosoid 964 nuclear dataset	Koenen et al. (2020)
***Serianthescalycina* Benth.** *	ERS11697309	Barrabé 1158 (NOU)	expanded mimosoid 977 nuclear dataset	Ringelberg et al. (2022)
***Serianthesgermanii* Guillaumin** *	SRR17180693	MacKee HS 5036 (L), L.2034754	GoFlag 408 dataset	This contribution
***Seriantheshooglandii* Fosberg** *	SRR17180692	Schodde R 2750 (L), L.2034739	GoFlag 408 dataset	This contribution
**Seriantheskanehiraevar.kanehirae (Ukall, Kumer - Palau)** *	SRR1718091	Demeulenaere E, PAL006	GoFlag 408 dataset	This contribution
***Serianthesmelanesica* Fosberg** *	SRR1718090	Drake DR; 256 (US); US2191202	GoFlag 408 dataset	This contribution
***Serianthesminahassae* (Koord.) Merrill & Perry** *	SRR1718089	Pullen R, 6484 (L); L.1995177	GoFlag 408 dataset	This contribution
***Serianthesnelsonii* (Håyun Lågu - Guam)** *	SRR1718088	Demeulenaere E, GUA002	GoFlag 408 dataset	This contribution
***Serianthesvitiensis* A. Gray** *	SRR1718087	Gardner RO, 6872 (US); US942100	GoFlag 408 dataset	This contribution
***Sphingaacatlensis* (Benth.) Barneby & J.W. Grimes**	ERS4812941	C.E. Hughes 2112 (FHO)	mimosoid 964 nuclear dataset	Koenen et al. (2020)
***Stryphnodendronpulcherrimum* (Willd.) Hochr.**	ERS4812942	L.P. de Queiroz 15482 (HUEFS)	mimosoid 964 nuclear dataset	Koenen et al. (2020)
***Viguieranthusglaber* Villiers**	ERS4812947	E. Koenen 325 (Z)	mimosoid 964 nuclear dataset	Koenen et al. (2020)
***Wallaceodendroncelebicum* Koord.** *	ERS11697328	Tim Flynn 7173 (NYBG)	expanded mimosoid 977 nuclear dataset	Ringelberg et al. (2022)

### ﻿DNA extraction, library preparation and enrichment

DNA extractions of the *Serianthes* samples for sequencing the GoFlag angiosperm 408 probe set followed the protocol of Breinholt et al. (2021a). Following bead clean-up, DNA was normalised and Illumina-compatible libraries were prepared following standard procedures (Breinholt et al. 2021a). Library construction, target enrichment and sequencing of *Serianthes* samples were done by RAPiD Genomics (Gainesville, Florida, U.S.A.) using protocols of Breinholt et al. (2021a). Target enrichment used the angiosperm version of the GoFlag 408 probe set (Breinholt et al. 2021a) that covers 408 conserved nuclear exons across 229 of the single- or low-copy genes identified by the 1KP transcriptome sequencing project (Leebens-Mack et al. 2019). All enriched samples were sequenced using an Illumina HiSeq 3000 (Illumina, San Diego, California, USA) with paired-end 100 base-pair reads.

### ﻿Data filtering and assembly

For the GoFlag 408 samples, we used a modified version of the iterative baited assembly pipeline of Breinholt et al. (2021a, b) to recover the targeted nuclear exon loci and the more variable flanking intron regions from enriched Illumina data. Our modified pipeline differed from the original pipeline in that: 1) reference sequences used in the *de novo* assembly of the loci were from 690 angiosperm samples extracted from the 1KP alignments of single copy nuclear loci (Leebens-Mack et al. 2019) corresponding to the 408 target regions; 2) we used 10 angiosperm genomes, rather than flagellate land plant genomes, to assess orthology; 3) to filter non-angiosperm contaminants, we performed a tBLASTx (Camacho et al. 2009) search against the respective angiosperm and flagellate land plant reference sequences for each locus. If a sequence’s best hit was not from an angiosperm, that sequence was removed as a potential contaminant. The pipeline outputs sequences for each locus. To minimise the possibility of including paralogs, we removed loci from a sample’s alignment when multiple sequences were recovered for a single locus alignment. For the eight *Serianthes* samples, we removed an average of 6.6% of loci due to presence of multiple sequences.

To recover sequences with as many shared loci as possible from the 964 and 997 gene *Mimobaits* datasets of Koenen et al. (2020) and Ringelberg et al. (2022), we downloaded raw reads for these samples from the NCBI Sequence Read Archive (SRA) database. We ran the same pipeline to recover sequences from as many of the GoFlag angiosperm 408 loci as possible. This resulted in 77 shared loci for 57 taxa, each containing the targeted exon and flanking regions. We excluded samples for which fewer than 10 GoFlag loci were recovered. Specimens with more than 72% gaps or ambiguities in the concatenated alignment were removed from gene alignments. The 72% threshold coincides with the gap/ambiguity value for *Falcataria*, a key taxon in our analysis that was inferred to be sister to *Serianthes* by Koenen et al. (2020). Other studies have applied similar (75%; Koenen et al. 2020) or more stringent (50%; Spillane et al. 2021) thresholds to account for compositional bias. Based on the 72% threshold, we retained 19 taxa of the Archidendron clade. By excluding taxa with fewer than 10 loci or more than 72% gaps or ambiguities, 43 of the 115 taxa in the original *Mimobaits* 964 nuclear dataset (Koenen et al. 2020; Table 2) and six taxa from the expanded mimosoid 997 gene dataset (Ringelberg et al. 2022; Table 2) were retained. We aligned sequences from these 49 species with seven *Serianthes* samples and one outgroup generated using the GoFlag angiosperm 408 dataset (Table 2) using MAFFT version 7.425 (Katoh and Standley 2013). The presence of indels in the flanking intron regions of the GoFlag target exons and the substantial variation in the amount of flanking sequence recovered from each sample resulted in regions of the alignment with nucleotide data from only one or a few samples. To reduce this missing data, we used a Perl script to eliminate any columns in the alignment of each locus that included fewer than ten nucleotides.

### ﻿Concatenated, gene tree and concordance analyses

A partitioned ML analysis of the concatenated multi-locus alignment was run in IQ-TREE (Nguyen et al.2015; Minh et al. 2020b). ModelFinder (Kalyaanamoorthy et al. 2017) was used to identify the best-fit substitution model for each locus. Ultrafast bootstrap approximations (UFBoot) were calculated to evaluate branch support in a single IQ-TREE run. ASTRAL-III (Zhang et al. 2018) was used to infer a species tree while accounting for possible incomplete lineage sorting amongst gene trees (Koenen et al. 2020). Each of the 77 gene trees was constructed using Maximum Likelihood analyses comparable to the partitioned analysis of the concatenated alignment. These gene trees served as input for the ASTRAL analysis to infer a species tree with local posterior probabilities (PP) as node support values. Polytomy tests (Sayyari and Mirarab 2018) to assess if polytomy null models could be rejected at a particular node (p < 0.05) were conducted in ASTRAL-III. Gene tree (dis)concordance analyses were performed in IQ-TREE to assess levels of gene tree conflict across the species tree (Chan et al. 2020; Minh et al. 2020a).

**Figure 3.**
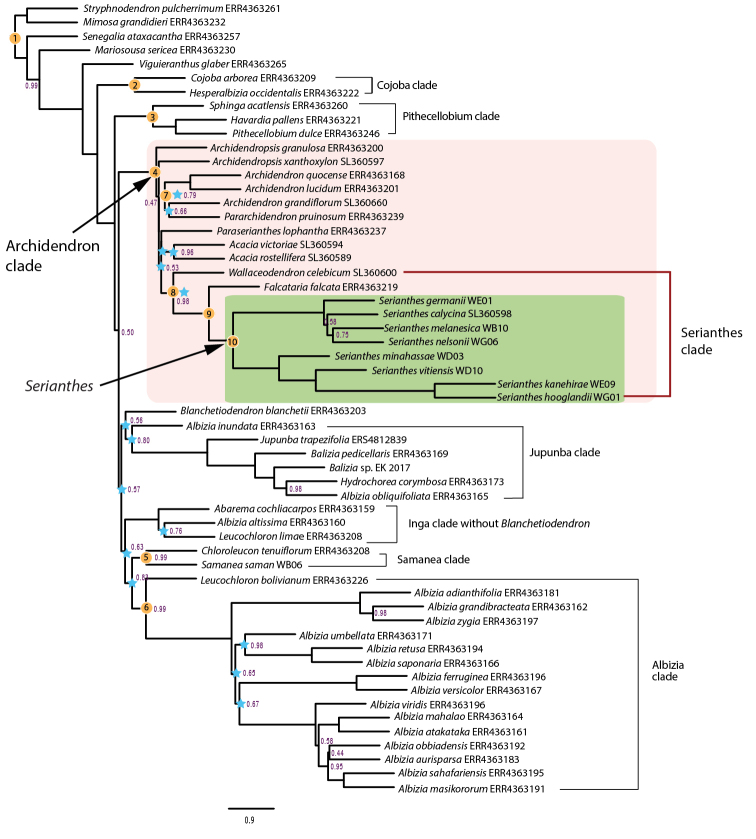
Phylogeny of the ingoid and Archidendron clades. ASTRAL species tree, based on 77 gene trees. Nodes of particular interest are labelled with numbered orange circles and are discussed in the text and Table 3. Unambiguously supported relationships shown with PP = 1 unless indicated at the nodes. Blue stars show nodes where a polytomy cannot be rejected by the data using the polytomy test (p ≤ 0.05). Clade names follow (Koenen et al. 2020), except for the Serianthes clade, which is newly recognised here.

PP values of 1 provided unambiguous support for each branch (Fig. 3, Table 3). Gene concordance factors (gCF, the percentage of gene trees containing a specific branch in the species tree) and site concordance factors (sCF, the percentage of alignment sites supporting that branch) were calculated (Minh et al. 2020a, Table 4; Stubbs et al. 2020). sCF values have a lower bound of 33% because they are based on a quartet-based approach to calculate the value at each node (Burbrink et al. 2020). Robustly or fully supported branches with high bootstrap values in the species tree can still show conflicting signals in the gene trees due to incomplete lineage sorting (ILS), hybridisation, inconsistent paralog retention in polyploids, introgression, model mis-specification and stochastic error inherent in sequencing techniques. New methods may help to elucidate these processes using target capture data from nuclear loci in the future (e.g. Morales-Briones et al. 2021; Tiley et al. 2021).

**Table 3.** Comparison of support values for individual nodes from concatenated analysis vs. gene tree analysis. BS and *p*-value (polytomy test) generated by concatenated analysis. BS, PP and *p*-value (polytomy test) generated by gene tree analysis.

ID	Name	Concatenated analysis		Gene Tree Analysis		
		BS	*p*-value	PP	BS	*p*-value
**1**	ingoid clade	100	0.000	1.000	100	0.000
**2**	Cojoba clade	100	0.009	1.000	100	0.000
**3**	Pithecellobium clade	NA	NA	1.000	100	0.000
**4**	Archidendron clade	100	0.270	1.000	100	0.000
**5**	Samanea clade	NA	NA	0.99	99	0.001
**6**	Albizia clade	100	0.000	0.99	100	0.011
**7**	*Archidendron* + *Pararchidendron*	100	0.000	0.79	100	0.285
**8**	Serianthes clade (*Wallaceodendron* + *Serianthes* + *Falcataria*)	100	0.000	0.980	100	0.056
**9**	*Falcataria* + *Serianthes*	100	0.000	1.000	100	0.000
**10**	*Serianthes*	100	0.000	1.000	100	0.000

The two gene discordance factors, gDF_1_ and gDF_2_, quantify the support for the two nearest-neighbour interchange partitions. The third gene discordance factor, gDF_P_ (“paraphyletic discordance factor”), calculates the support for all possible topologies (Minh et al 2020a; Thomas et al. 2021). There are three possible quartets around each branch it supports (based on sites), the first one is the sCF, the second one sDF_1_ calculates the support amongst sites for alternative quartets and sDF_2_ calculates the support for a second alternative arrangement (Minh et al. 2020a; Thomas et al. 2021). The sum of sCF, sDF_1_ and sDF_2_ values is 100%. Correlations between concordance factors and support values were visualised in R (R Core Team 2021). The pipeline to run the analyses in IQ-TREE, ASTRAL-III and the visualisation of relationships between concordance factors in R followed Lanfear (2018) and Matschiner (2020).

**Table 4.** Comparison of concordance, discordance factors and branch lengths calculated in IQ-TREE for individual nodes in the Mimosoid phylogeny.

ID	Name	Concordance Analysis							
		gCF	sCF	gDF_1_	gDF_2_	gDF_P_	sDF_1_	sDF_2_	BranchL
**1**	ingoid clade	53.61	51.32	18.84	17.39	10.14	22.51	26.18	0.474
**2**	Cojoba clade	52.46	65.79	3.28	3.28	40.98	14.86	19.35	0.743
**3**	Pithecellobium clade	40.00	64.25	0.00	0.00	60.00	16.620	19.13	0.760
**4**	Archidendron clade	21.33	69.59	0.00	0.00	78.670	15.69	14.72	0.748
**5**	Samanea clade	29.410	45.54	5.88	4.41	60.29	26.72	27.74	0.283
**6**	Albizia clade	25.37	51.25	5.97	2.99	65.67	22.56	26.20	0.278
**7**	*Archidendron* + *Pararchidendron*	9.86	46.67	0.00	2.82	87.32	25.78	27.56	0.118
**8**	Serianthes clade (*Wallaceodendron* + *Serianthes* + *Falcataria*)	16.92	58.08	0.00	3.80	80.00	19.73	22.19	0.241
**9**	*Falcataria* + *Serianthes*	44.83	69.28	5.17	5.17	44.83	12.31	18.41	0.707
**10**	*Serianthes*	46.97	65.60	12.12	13.64	27.27	17.13	17.27	0.480

## ﻿Results

### ﻿Assembly

The matrix comprised 77 exons and flanking regions for 57 taxa (Table 2) and was 115,160 bp in length. Of the 45,600 variable sites, 15,210 were parsimony-informative and 30,390 were singleton sites.

### ﻿Phylogenetic inference and quantification of gene tree and site conflicts

**Figure 4.**
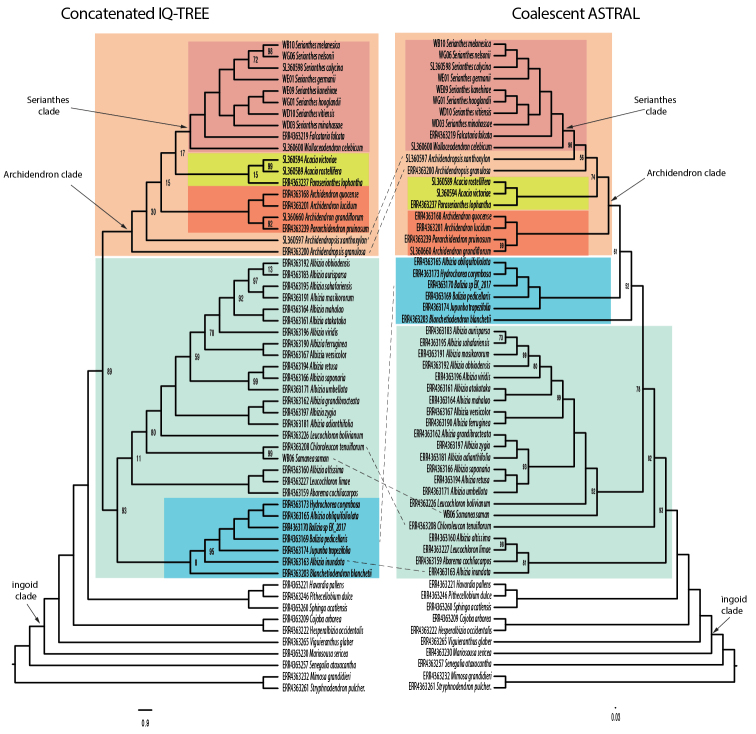
Backbone phylogeny of the ingoid clade. Comparison between the concatenated ML tree (left) and ASTRAL partition tree analysis (right). Bootstrap values < 100% are indicated below the nodes. Major clades in the IQ-tree and phylogenetic grades in the ASTRAL tree are shown in colour blocks with the incongruences between them indicated by dashed lines.

The ASTRAL species tree and the concatenated ML tree from IQ-TREE have largely similar Archidendron clade topologies (Fig. 4), with higher support values (BS and PP) in the ASTRAL tree compared to the concatenated ML analysis (Figs 3, 4). Although there are topological differences between the ASTRAL species tree and the concatenated ML analysis from IQ-TREE outside of the Archidendron clade, the ASTRAL tree is better resolved.

**Figure 5.**
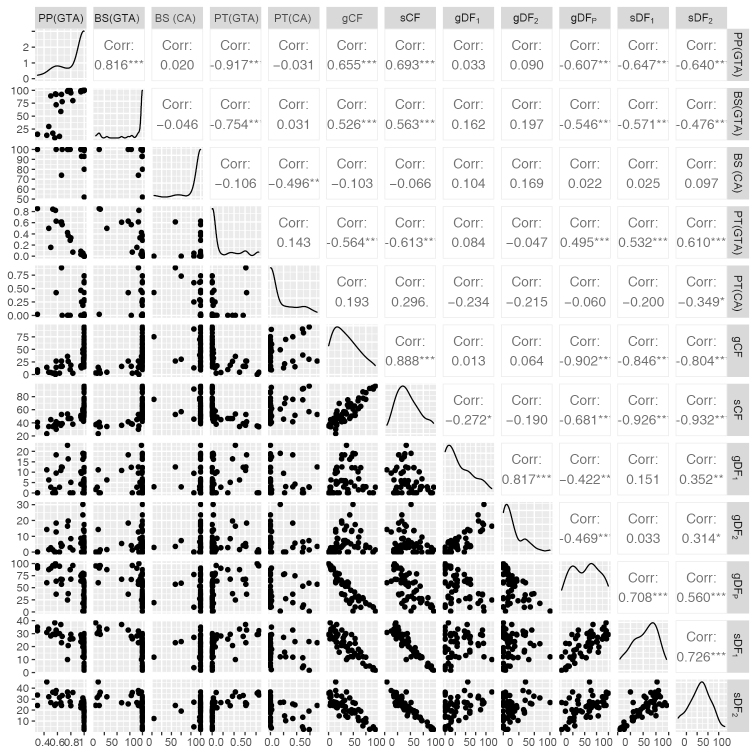
Scatter plots from gene discordance analysis. The graphs show the relationships between PP (gene tree analysis [GTA]), BS (GTA), BS (concatenated analysis [CA]), polytomy test [PT] (GTA), PT (PA), gene concordance factor (gCF), site concordance factor (sCF), gene discordance factors (gDF1, gDF2), gene discordance factor (P stands for paraphyly) (gDFP) and site discordance factors (sDF1, sDF2). The strength and direction of correlations (r) between variables are described as follows: r = -1, perfect negative relationship; -1 < r ≤ -0.70, strong negative relationship; -0.70 < r ≤ -0.50, moderate negative relationship; -0.50 < r ≤ -0.30, weak negative relationship; -0.30 < r < 0.30, no relationship; 0.30≥ r < 0.50, weak positive relationship; 0.50 ≥ r < 0.70, moderate positive relationship; 0.70 ≥ r < 1, strong positive relationship; r = 1, perfect positive relationship.

**Figure 6.**
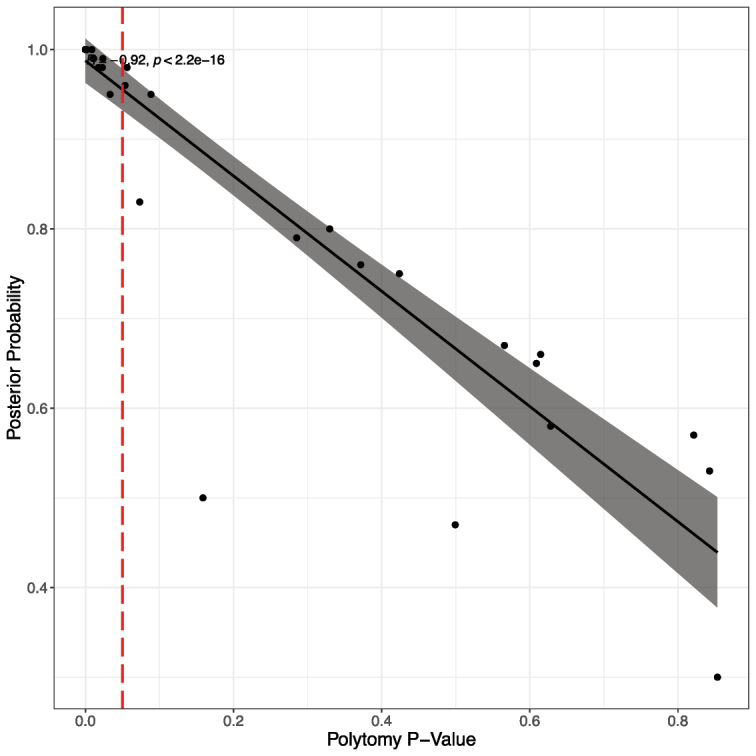
Pearson correlation showing the relationship between polytomy *p*-value and PP (gene tree analysis). We visualise the branches for which the polytomy null model could be rejected, based on the ASTRAL polytomy test at *p* < 0.05, indicated by the red dashed line.

Local posterior probability values and polytomy *p*-values of the ASTRAL species tree analysis are strongly negatively correlated (*r* = -0.917; Figs 5, 6; Lanfear 2018). Fig. 6 shows that almost all the nodes for which the polytomy null model was rejected (*p* < 0.05) have high local posterior probability values.

**Figure 7.**
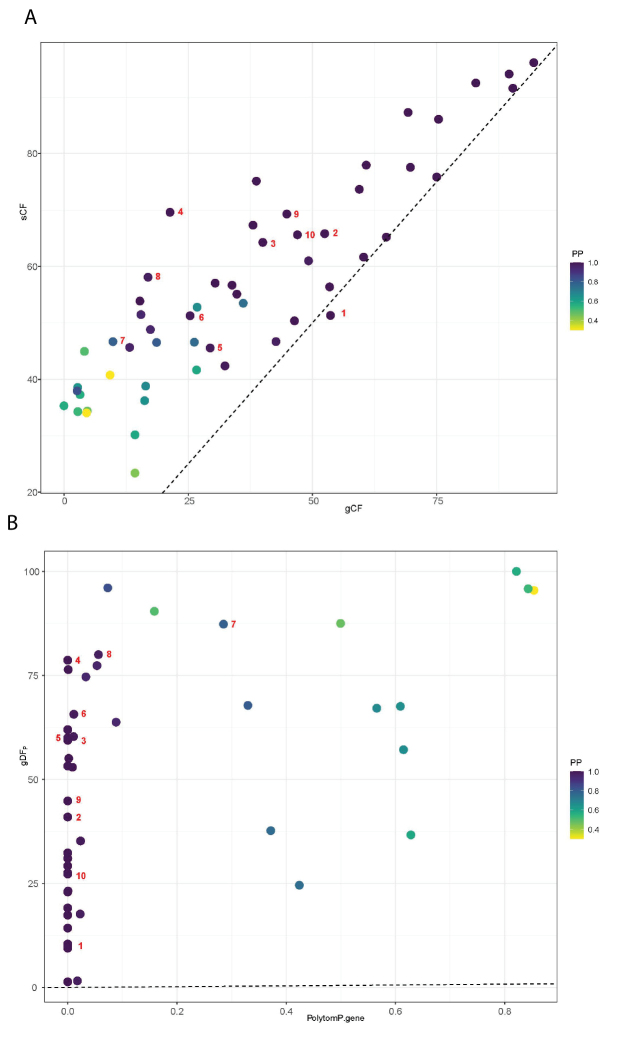
**A** scatter plot showing PP values and the relationship to gene concordance factors (gCF) and site concordance factors (sCF) (gene tree analysis). The red numbers coincide with the branch numbers of Table 4 and Fig. 3**B** scatter plot showing p-value (polytomy test) and the relationship to gene discordance factors (paraphyly) (gDFP). Points show each bipartition in the full dataset phylogeny, with red numbers coinciding with the branch numbers in Fig. 3 and Table 4.

The tree topology is described, based on the ASTRAL analysis focusing on 10 nodes for which the polytomy null model could be rejected (numbered in Fig. 3; Table 3). Bootstrap values and polytomy test *p*-values of the concatenated analysis are listed in Table 3. The gCF and sCF values showed a strong positive correlation (*r* = 0.888; Fig. 5) and high PP values mostly coincide with medium to high gCF and sCF values (Fig. 7A).

Clade names used in this manuscript follow the mimosoid clade classification of Koenen et al. (2020). The ingoid clade (sensu Koenen et al. 2020) (node 1) is well supported by high PP and BS values and the null hypothesis of the node being replaced by a polytomy is rejected (*p* = 0.001). Low sDF_1_, sDF_2_, gDF_1_, gDF_2_, gDF_P_ and medium sCF provide confidence that this split is well supported (Table 4, Fig. 7B). The backbone of the ingoid clade is only partly resolved. The Cojoba clade (node 2), Pithecellobium clade (node 3), Archidendron clade (node 4), Samanea clade (node 5) and Albizia clade (node 6) were all recovered with high PP and BS values and their polytomy null models were rejected (*p* = 0.001) (Fig. 3; Table 3). We recovered *Albizia* and *Leucochloron* as polyphyletic. The relationship between the Jupunba clade and the Inga clade remained unresolved along the backbone of the ingoid clade (Fig. 3). Note that, in our analyses, the ingoid clade does not include representatives from the Calliandra and Zapoteca clades. The gCF and sCF values are medium to high for all selected clades with the exception of the Archidendron clade (node 4), the Samanea clade (node 5), the Albizia clade (node 6) and the Wallaceodendron + Serianthes + Falcataria clade (node 8; Fig. 3; Table 3). gDFs and sDFs estimates are low, while values of gDF_P_ are rather high for most of the numbered clades, except the ingoid clade (node 1), the Cojoba clade (node 2), the Falcataria + Serianthes clade (node 9) and the Serianthes clade (node 10) (Fig. 3, Table 3), which also had longer branch lengths (Table 4). The concatenated analysis retained unresolved relationships across the ingoid backbone, except for the Pithecellobium clade (Fig. 3).

Our analyses strongly support the monophyly of the Archidendron clade (PP = 1, BS = 100), with a polytomy rejected at this node in the gene tree analysis (*p* = 0.001) (node 4 on Fig. 3, Table 3). The concordance analysis for this node provided a gCF value of 21.33% and sCF value of 69.59%. Discordance analysis returns low sDF_1_ and sDF_2_ values of 15.69% and 14.72%, respectively and low gDF_1_ and gDF_2_ values of 0% and high gDF_P_ of 78.67%. Taking the low support from the gene concordance factors and gene discordance factors into account, it is important to note that the polytomy in the concatenated analysis phylogeny was not rejected (*p* = 0.270) in the ASTRAL analysis.

Furthermore, our analyses support the sister relationship of *Serianthes* and *Falcataria* with unambiguous BS and PP support, with a high gCF value of 44.83% and a sCF value of 69.28% (node 9) (Fig. 3; Tables 3, 4). The polytomy test for this node is rejected at *p* < 0.001 (Table 3). The gDF_P_ value is 44.83%, while low gDF_1_ (5.17%), gDF_2_ (5.17%), sDF_1_ (12.31%) and sDF_2_ (18.41%) values are recovered. *Wallaceodendron* is resolved as sister to the Serianthes + Falcataria clade (node 8) (Fig. 3; Tables 3, 4). For this relationship, we also find unambiguous BS and PP for both gene tree and concatenated analyses and a gCF value of 16.92% and sCF value of 58.08%. The polytomy test is not rejected at *p* < 0.056 and gDF_P_ (80.00%) is high, but the gDF (0% and 3.8%) and sDF (19.73%, 22.19%) are very low. Based on our results, we informally name the Serianthes clade (node 8, Fig. 3) to include the genera *Falcataria*, *Serianthes* and *Wallaceodendron*.

The ASTRAL species tree topology, using a representative sample of eight species of *Serianthes*, confirmed its monophyly (node 10) with unambiguous BS and PP support in the gene tree analysis (Fig. 3; Table 3). The polytomy test for this node was rejected (*p* < 0.001) and a high gCF value of 46.97% and an sCF value of 65.60% coincided with a low gDF_P_ value of 27.27%. The discordance analysis further showed low gDF1 (12.12%), gDF_2_ (13.64%), sDF_1_ (17.13%) and sDF_2_ (17.27%) values. We identify two well-supported subclades within *Serianthes*. The first one comprises taxa from Malesia, Papuasia and southern Micronesia (*S.minahassae* (Koord.) Merrill & Perry, *S.vitiensis* A. Gray, *S.kanehirae* Fosberg, *S.hooglandii* Fosberg), while the other clade unites all taxa from Polynesia and northern Micronesia (*S.germanii* Guillaumin, *S.calycina* Benth., *S.melanesica* Fosberg, *S.nelsonii* Merr.).

Our analyses also confirm the close relationship between *Archidendron* and *Pararchidendron* (node 7; Fig. 3; Table 3). This topology did not reject the polytomy at *p* = 0.285, but has a BS = 100% and PP = 0.79 for the gene trees and a BS = 100 for the concatenated analyses and a gCF value of 9.86% and a sCF value of 46.67%. The gDFP (87.32%) value is very high and the gDF_1_, gDF_2_ (0.00% and 2.82%) and sDF_1_, sDF_2_ (25.78%, 27.56%) values were low.

Low gDF_P_ values were found for the tips of the generic clades, while high gDF_P_ values were found along the backbone of the ingoid and Archidendron clades. Polytomies were rejected for the tips of the clades, for instance, in the Albizia and Serianthes clades, which are accompanied by high gCF and sCF, low gDFs and sDFs and low gDF_P_ values.

## ﻿Discussion

Our study provides the first molecular evidence that *Serianthes*, as delineated by Nielsen et al. (1984a), is monophyletic (node 10) (Fig. 3; Tables 3, 4). Diagnostic features of *Serianthes* include bipinnately compound leaves with alternate sessile leaflet insertion, branched axillary corymbiform panicles and woody indehiscent pods (Fosberg 1960; Nielsen et al. 1984b), as opposed to bipinnately compound leaves with opposite leaflets and dehiscent pods in *Wallaceodendron* and *Falcataria*. The spiciform racemes of *Wallaceodendron* are solitary, while they are compound in *Falcataria*. Nielsen et al. (1984a) also commented on differences in pollen morphology between *Serianthes* and other genera in the Archidendron clade (Table 5), whereby the tectum of *Wallaceodendron* and *Serianthes* (except for subgenus Serianthessect.Minahassae) is perforated by non-isometric channels, as compared to isodiametric channels in the other genera of the Archidendron clade (Nielsen et al. 1984a). Further research is needed to evaluate the taxonomic significance of pollen exine stratification across the Archidendron clade as a whole.

**Table 5.** Morphology of *Serianthes*, *Falcataria* and *Wallaceodendron*, based on Fosberg (1960), Nielsen (1992), Nielsen et al. (1983, 1984a, b) and Verdcourt (1979).

	*Wallaceodendron*	*Falcataria*	*Serianthes*
**Inflorescence**	Solitary axillary unbranched spiciform raceme	Unbranched elongated raceme	Umbel, raceme or panicle composed of pedunculate spikes, pedunculate racemes or 1–4 flowered glomerules
**Pod**	Dehiscent, unwinged	Dehiscent, narrow wing	Indehiscent, unwinged
**Epicarp**	Chartaceous to woody	Chartaceous to woody, dehiscent, narrow wing	Thin, coriaceous, chartaceous to woody
**Endocarp**	Membranaceous to chartaceous	Chartaceous	Parchment-like, woody
	Endocarp forms a papery envelope around each seed, which is the basic dispersal unit		
**Germination**	Not known	Epigeal	Epigeal
**First two foliar leaves of the seedling**	Not known	Opposite and bipinnate	Opposite and bipinnate
**Leaf phyllotaxy**	Spiral	Alternate	Alternate
**Leaflet insertion**	Opposite	Opposite	Alternate
**Pollen exine**	Tectum perforated by non-isometric channels	Tectum perforated by isometric parallel channels	Tectum perforated by non-isometric channels (except in subgenus Serianthessect.Minahassae)

The close relationship amongst *Serianthes*, *Falcataria*, and *Wallaceodendron* as suggested by Nielsen et al. (1983), based on morphology, is corroborated by our phylogenomic analysis and this group is here referred to as the Serianthes clade (Fig. 3). The centre of diversity of the Serianthes clade is the Malesian and Papuasian region. Of this clade, *Serianthes* is the only genus with Pacific Island representatives, while *Falcataria* is the only genus occurring in Australia. *Serianthes* is the most widespread, most likely because of its indehiscent pods, which are dispersed via ocean currents (Demeulenaere and Ickert-Bond 2022).

The monophyly of *Serianthes* and the relationships within the Serianthes clade (nodes 8, 9 and 10; Fig. 3; Tables 3, 4) received full support, suggesting that the alignments were informative and provided a clear signal for these relationships. Nielsen et al. (1983, 1984a) postulated that *Paraserianthesfalcataria* (now *Falcatariafalcata*) is closely related to *Serianthes*, observing that the bracts of the two are large and concave and have barely distinguishable wood anatomy (Nielsen et al. 1983). *Serianthes* and *Falcataria* also share opposite and bipinnate seedling leaves, while mature leaves of *Serianthes*, in contrast to *Falcataria*, have alternate leaflet insertion (Table 5). This phylogenomic study provides the first evidence of two deeply-divergent and robustly-supported subclades within *Serianthes*, one comprising *S.germanii*, *S.calycina*, *S.melanesica* and *S.nelsonii* and the second *S.minahassae*, *S.hooglandii*, *S.vitiensis* and *S.kanehirae*. The placements of other *Serianthes* species within these subclades and how they correspond to the classification of subgenera and sections from Nielsen et al. (1984a) will require more complete taxon sampling.

*Serianthes* and *Falcataria* are sister genera in our phylogenomic study (Fig. 3), corroborating the results of Ringelberg et al. (2022), but not Brown et al. (2022). Both genera have alternate leaves, while *Wallaceodendron* has leaves that are spirally arranged (Sosef et al. 1998). *Wallaceodendron* was recovered as sister to *Serianthes* + *Falcataria* in our study (Fig. 3). Fosberg (1960) treated these three genera as distinct, noting that, while the flowers and the fruits of *Wallaceodendron* and *Serianthes* are very similar, *Serianthes* has flowers arranged in panicles, rather than racemes in *Wallaceodendron*, the pods of *Serianthes* are indehiscent, compared to the dehiscent pods of *Wallaceodendron* (tardily dehiscent) and *Falcataria*, and *Wallaceodendron* and *Falcataria* have strictly opposite leaflets as opposed to alternate leaflets in *Serianthes* (Fosberg 1960; Kanis 1979; Nielsen et al. 1983). This combination of inflorescence, leaf, and fruit dehiscence differences supports recognition of three distinct genera.

**Figure 8.**
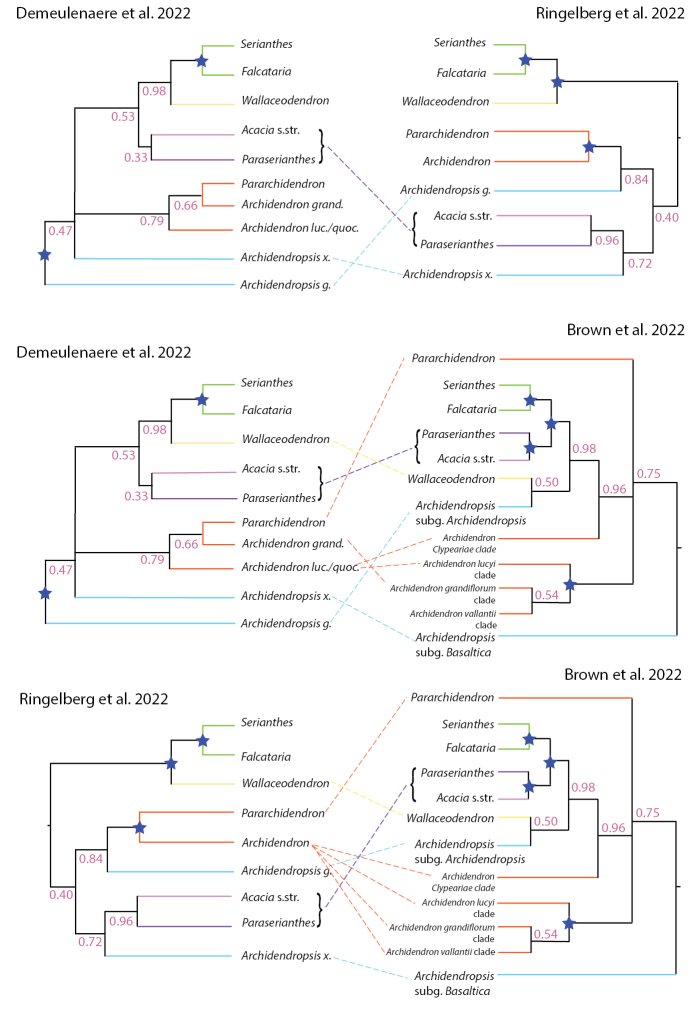
Comparison of relationships of the Archidendron clade recovered by different authors. Colour schemes follow those in Fig. 2. The branches that are fully supported (either by PP = 1.00 or BS = 100%) are indicated by blue stars and discordant placements of genera are indicated by dashed lines. The following abbreviations are used: *Archidendrongrandi*. = *Archidendrongrandiflorum*, *Archidendronluc*. = *Archidendronlucidum*, *Archidendronquoc*. = *Archidendronquocense*, *Archidendropsisg.* = *Archidendropsisgranulosa* and *Archidendropsisx.* = *Archidendropsisxanthoxylon*.

Our phylogeny suggests that *Pararchidendron* is nested within *Archidendron*, rendering *Archidendron* paraphyletic (Fig. 8) as also found by Brown et al. (2022). Many nodes along the backbone of the Archidendron clade remain poorly resolved (Fig. 8). The sister relationship of *Paraserianthes* and *Acacia* s.s. agrees with Brown et al. (2022) and Ringelberg et al. (2022). A recent phylogeny of legumes as a whole found full support for the sister relationship between the monophyletic *Acacia* s.s. and a clade containing *Falcataria*, *Pararchidendron* and *Archidendron* (Zhao et al. (2021), but this study did not include *Paraserianthes*. The position of *Archidendropsis* within the Archidendron clade remains uncertain, but the genus is not supported as monophyletic in our analyses (Fig. 8) – see Brown et al. (2022). Increased taxon sampling with phylogenomic data is needed to resolve the relationships of *Archidendron*, *Archidendropsis* and *Pararchidendron*.

Conflicting topologies amongst sites and genes occurred where nodes showed low sCF and gCF values (nodes 4, 5 and 8 in Fig. 3; Table 4), which are indicative of discordant signals between gene trees. This was also shown by the short internode distances (expressed in coalescent units) at these branches in our phylogeny (Fig. 3). High gDF_P_ values coincided with short branches and likely indicate rapid lineage divergence (evolutionary radiation) and/or ILS (nodes 3, 5, 6, 7 and 8; Fig. 3; Table 4). This is consistent with the large putative hard polytomy in the ingoid clade discovered by Koenen et al. (2020), which likely represents a rapid radiation of a set of six or seven lineages. The Archidendron clade (node 4 in Fig. 3; Tables 3, 4) is one of the lineages derived from that putative hard polytomy along part of the backbone of the ingoid clade. The gene tree analysis provided high node support (PP = 1, BS = 100) and a high sCF value of 69.59% supporting the obtained tree topology at this node. The observed low discordance factor values (between 0 and 14.72%; Minh et al. 2020a; Thomas et al. 2021), however, indicated notable conflicts between gene concordance and discordance factors. The concordance analysis provided high gDF_P_ values of 78.67%, indicating that the gene trees lacked a clear signal (Minh et al. 2020a; Thomas et al. 2021). The fact that high PP and BS values coincided with low gCF values illustrates that classical node support measures, such as PP and BS, do not capture all aspects of variation in large phylogenomic datasets (Brower 2006, 2018; Thomas et al. 2021).

## ﻿Conclusions

Sequence capture (Grover et al. 2012) provides a cost-effective way to generate hundreds of informative markers for plant phylogenomics that can be used across taxonomic scales (Zimmer and Wen 2015), including recent radiations of species and in intraspecific phylogeography (Nicholls et al. 2015). There is growing interest in combining data from different probe sets and, particularly, the merger of data from universal probe sets with data from clade-specific probes (e.g. Hendriks et al. 2021). Our study shows that the merger of data from different probe sets can yield enough overlapping loci to resolve intergeneric relationships. Our ingoid dataset increased resolution in the ingoid and Archidendron clades and generated a well-supported phylogeny, representing the evolution of unlinked markers across the genome. In many cases, the concordance analysis provided a new perspective on bootstrap values, local posterior probability support levels and polytomy tests, which may be inflated in large, concatenated alignments (Minh et al. 2020a; Thomas et al. 2021). Our analyses provide robust evidence for: (1) the monophyly of *Serianthes* and two main lineages within the genus; (2) the Serianthes clade, which sets the stage for future biogeographic analysis of this clade and highlights the close sister relationship between *Wallaceodendron* and *Serianthes* + *Falcataria*; (3) rapid radiations across the backbones of the ingoid and Archidendron clades, which may be difficult to resolve without extensive genomic data; the concordance analysis clarified the interpretation of phylogenetic relationships; in particular, we found limited gene conflicts near the tips of the Archidendron clade, but an increase in discordance at the base of the clade; and (4) the utility of the polytomy test to further evaluate if gene tree discordance affects node support values. Continued sampling and sequencing of *Serianthes* species and other genera in the Archidendron clade are necessary to fully evaluate the generic delimitation and relationships within the Archidendron clade.
